# Molecular epidemiology analysis of fowl adenovirus detected from apparently healthy birds in eastern China

**DOI:** 10.1186/s12917-022-03545-5

**Published:** 2023-01-09

**Authors:** Qingye Zhuang, Suchun Wang, Fuyou Zhang, Chenglong Zhao, Qiong Chen, Ran Zhao, Pin Guo, Lei Ju, Jinping Li, Guangyu Hou, Xiaoying Chen, Fuliang Sun, Kaicheng Wang

**Affiliations:** 1 China Animal Health and Epidemiology Center, 369 Nanjing Road, Qingdao, Shandong Province China; 2 Shandong Vocational Animal Science and Veterinary College, Weifang, Shandong Province China; 3 Xiamen Agricultural Product Quality and Safety Testing Center, Xiamen, Fujian Province, China; 4 Yanbian University, Yanbian, Yanji, Jilin Province 133002 China; 5 Key Laboratory of Animal Biosafety Risk Prevention and Control (South), Ministry of Agriculture and Rural Affairs, Qingdao, P.R. China

**Keywords:** Fowl adenoviruses, Apparently healthy birds, Epidemiological survey, Living bird markets

## Abstract

**Background:**

Fowl adenovirus is of major concern to the poultry industry worldwidely. In order to monitor the prevalent status of Fowl adenovirus in China, a total of 1920 clinical samples from apparently healthy birds in the 25 sites of poultry flocks, Slaughterhouse and living bird markets from 8 provinces in eastern China were collected and detected by PCR, sequencing, and phylogenetic analysis.

**Results:**

The epidemiological survey showed that Fowl adenoviruses were detected in living bird markets, and circulating in a variety of fowl species, including chickens, ducks, goose and pigeons. Among the 1920 clinical samples, 166 samples (8.65%) were positive in the fowl adenovirus PCR detection. In this study, totally all the 12 serotypes (serotypes of 1, 2, 3, 4, 5, 6, 7, 8A, 8B, 9, 10 and 11) fowl adenoviruses were detected, the most prevalent serotype was serotype 1. Phylogenetic analysis indicated that 166 FAdVs of 12 serotypes were divided into 5 fowl adenovirus species (*Fowl aviadenovirus A, B, C, D, E*).

**Conclusions:**

In the epidemiological survey, 8.65% of the clinical samples from apparently healthy birds were positive in the fowl adenovirus PCR detection. Totally all the 12 serotypes fowl adenoviruses were detected in a variety of fowl species, which provided abundant resources for the research of fowl adenoviruses in China. The newly prevalent FAdV serotypes provides valuable information for the development of an effective control strategy for FAdV infections in fowls.

## Background

Fowl adenoviruses (FAdV) belong to genus *Aviadenovirus* in the family *Adenoviridae*, which resulting in huge economic losses to the poultry industry worldwide [1, 2]. FAdV is a double stranded DNA viruses, with a genome of 43–45 kb in size and a diameter of 70–100 nm [3]. Serologically different FAdV types are classified into five species (*Fowl aviadenovirus A-E*) [2, 4]. FAdVs are further classified into 12 serotypes (FAdV-1 to 8a and 8b to 11) by cross-neutralization tests [2, 5]. FAdV-2, FAdV-7, FAdV-8a, FAdV-8b and FAdV-11 can cause inclusion body hepatitis (IBH) [1, 2, 4, 6], and FAdV-4 can result in hepatitis hydropericardium syndrome (HHS) [2, 6].

FAdV can cause severe immunosuppression in infected birds. Clinical cases of FAdV infection have been widely reported in avian populations worldwide, and multiple FAdV strains have been isolated from dead or sick animals [1, 2, 7–10]. In China, FAdV-4 was considered as the dominant serotype in recent years [7, 11–13], which has resulted in huge economic losses in poultry industry in China since 2015 [11]. Several outbreaks caused by FAdV-4 was reported in China [4, 7, 11–14]. FAdV-8a and FAdV-8b were also the circulating serotypes in China between 2007 to 2014 and 2015 to 2018 [11, 14]. But, FAdV-11 was the predominant serotype in some regions in China from 2007 to 2014, according to the previous report [14]. FAdV-1 [1], FAdV-2 [15], FAdV-7 [4] and FAdV-10 [15] were also identified in China.

In this study, a total of 1920 clinical samples were collected from apparently healthy birds in 25 poultry flocks, Slaughterhouse and living bird markets (LBMs) from 8 provinces in eastern China. This is a comprehensive survey from apparently healthy fowls. FAdVs were detected and serotyped to investigate the prevalence of FAdV and analyze the genetic epidemiology.

## Results

### Epidemiological analysis

**Fig. 1:**
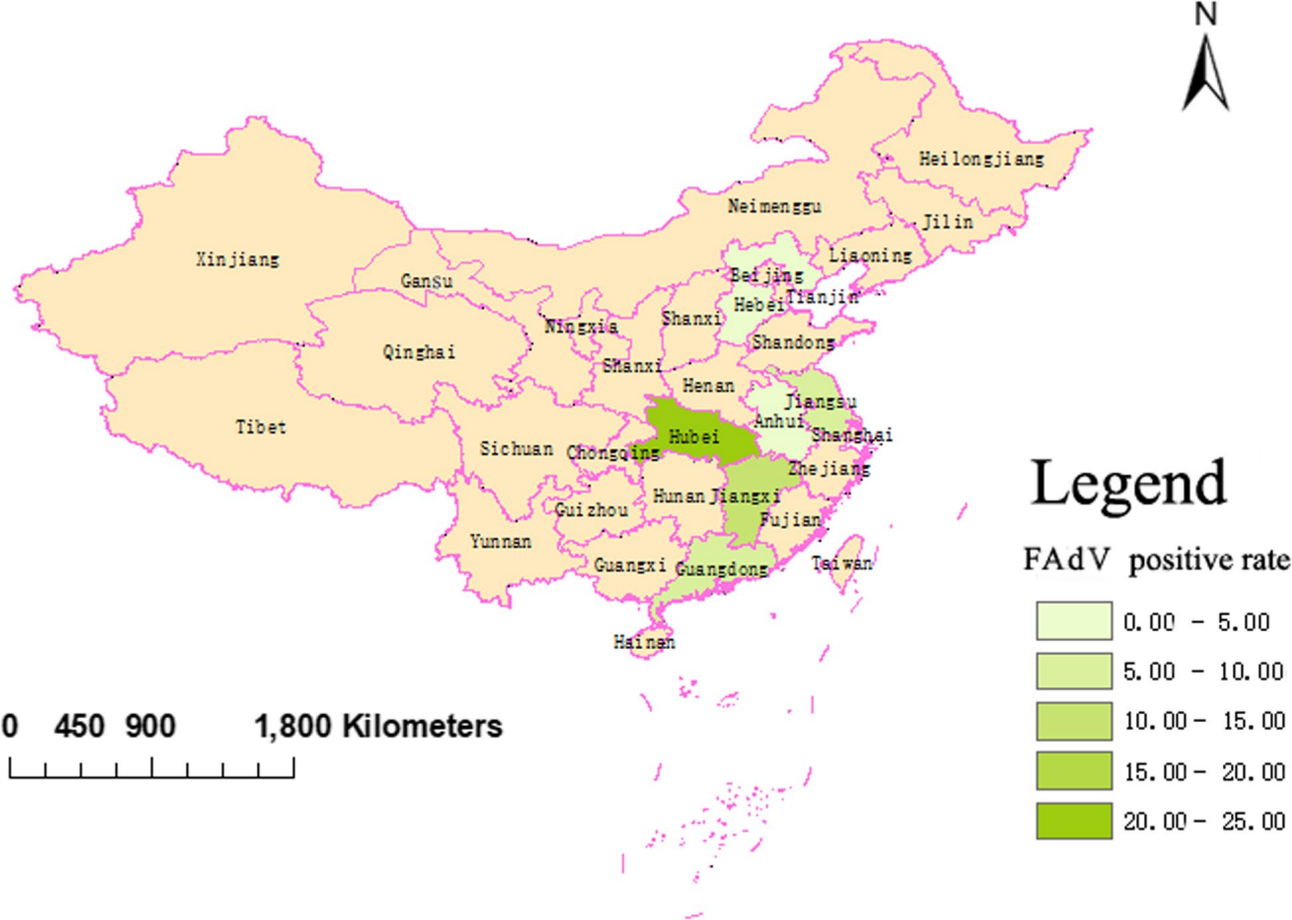
Geographical distribution of FAdVs circulating in the 8 provinces (Hubei, Jiangxi, Shanghai, Guangxi, Jiangsu, Guangdong, Anhui, Hebei) of China

Among the 1920 clinical samples, 166 samples (8.65%) were positive in the FAdV PCR detection. Spatial analysis showed that, except Hebei province, FAdVs were detected in all the other 7 provinces, including Hubei, Jiangxi, Shanghai, Guangxi, Jiangsu, Guangdong, Anhui (Fig. 1). The FAdV infection rate in Huhei province is the highest (23.75%). The details were showed in Table 1. All the FAdVs were detected from LBMs. All the samples collected from large-scale farms were negative in PCR detection. In the samples collected from 5 kinds of fowl, FAdV was detected in samples of chicken (13.27%), goose (2.50%), duck (0.48%) and pigeon (0.43%), while negative in the samples of partridge.

**Table 1:** The number and distribution of the 12 FAdV serotypes detected in the 8 provinces

Serotype	Province								Number of FAdV positive sample in total	Percentage of different serotyps in the detected FAdV (%)
	Hubei	Jiangxi	Shanghai	Guangxi	Jiangsu	Guangdong	Anhui	Hebei		
1	6	4	10	7	2	1	0	0	30	18.07
2	10	9	3	1	1	2	0	0	26	15.66
3	10	1	2	3	6	1	4	0	27	16.27
4	17	4	2	1	0	4	0	0	28	16.87
5	0	0	1	1	1	1	2	0	6	3.61
6	0	1	0	0	0	0	0	0	1	0.60
7	3	1	3	2	1	0	1	0	11	6.63
8A	1	6	2	0	0	1	0	0	10	6.02
8B	4	1	0	0	2	0	1	0	8	4.82
9	0	0	0	0	1	0	0	0	1	0.60
10	0	0	0	3	2	2	1	0	8	4.82
11	6	0	1	0	0	1	2	0	10	6.02
Number of FAdV positive samples in total	57	27	24	18	16	13	11	0	166	/
Number of screened samples	240	240	240	240	240	240	240	244	1920	/
FAdV infection rate (%)	23.75	11.25	10.00	7.50	6.67	5.42	4.58	0.00	8.65	/

### Molecular serotype identification of FAdVs

Total 12 FAdV serotypes were detected in the 1920 clinical samples. The hexon gene fragment sequences of the FAdVs were submitted to the GenBank database, National Center for Biotechnology Information and were assigned the accession numbers of ON502449-ON502604, and ON462358-ON462367. In the detected 166 FAdVs, the most prevalent serotypes were serotype 1 (30/166, 18.07%), serotype 4 (28/166, 16.87%), serotype 3 (27/166, 16.27%), serotype 2 (26/166, 15.66%) (Table 1). These 4 serotypes were accounting for 66.87% of all the detected FAdVs. All the 166 FAdV strains inferred the 12 serotypes were likely to be circulating in chickens. Besides in chicken samples, serotype 4 FAdVs were detected in duck, goose and pigeon samples, while serotype 8B FAdV was detected in duck samples. The FAdV serotype distribution in the 8 provinces and 5 kinds of fowls was showed in Tables 1 and 2, respectively.

**Table 2:** The number and distribution of the 12 FAdV serotypes detected in 5 kinds of poultry

Serotype	Animal					Number of FAdV positive sample in total
	Chicken	Goose	Duck	Pigeon	Partridge	
1	30	0	0	0	0	30
2	26	0	0	0	0	26
3	27	0	0	0	0	27
4	25	1	1	1	0	28
5	6	0	0	0	0	6
6	1	0	0	0	0	1
7	11	0	0	0	0	11
8A	10	0	0	0	0	10
8B	7	0	1	0	0	8
9	1	0	0	0	0	1
10	8	0	0	0	0	8
11	10	0	0	0	0	10
Number of FAdV positive sample in total	162	1	2	1	0	166
Number of screened samples	1221	40	419	235	5	1920
FAdV infection rate (%)	13.27	2.50	0.48	0.43	0.00	8.65

In 166 cases, 18.07% (30/166) of the isolates were related to *FAdV-A* (including serotype 1), and 3.61% (6/166) of the isolates were identified as *FAdV-B* (including serotype 5), and 21.69% (36/166) of the isolates were identified as *FAdV-C* (including serotypes 4 and 10), and 38.55% (64/166) of the isolates were identified as *FAdV-D* (including serotypes 2, 3, 9, and 11), and 18.07% (30/166) of the isolates were identified as *FAdV-E* (including serotypes 6, 7, 8A and 8B). The prevalent rate of circulating species was showed in Table 3.

**Table 3:** The prevalence rate of circulating species and serotypes in this study

^Species^	^Serotype^												Total	Rate
	1	2	3	4	5	6	7	8A	8B	9	10	11		
**A**	30	0	0	0	0	0	0	0	0	0	0	0	30	**18.07%**
**B**	0	0	0	0	6	0	0	0	0	0	0	0	6	**3.61%**
**C**	0	0	0	28	0	0	0	0	0	0	8	0	36	**21.69%**
**D**	0	26	27	0	0	0	0	0	0	1	0	10	64	**38.55%**
**E**	0	0	0	0	0	1	11	10	8	0	0	0	30	**18.07%**
**Total**	**30**	**26**	**27**	**28**	**6**	**1**	**11**	**10**	**8**	**1**	**8**	**10**	**166**	

### Phylogenetic analysis of FAdVs

**Fig. 2:**
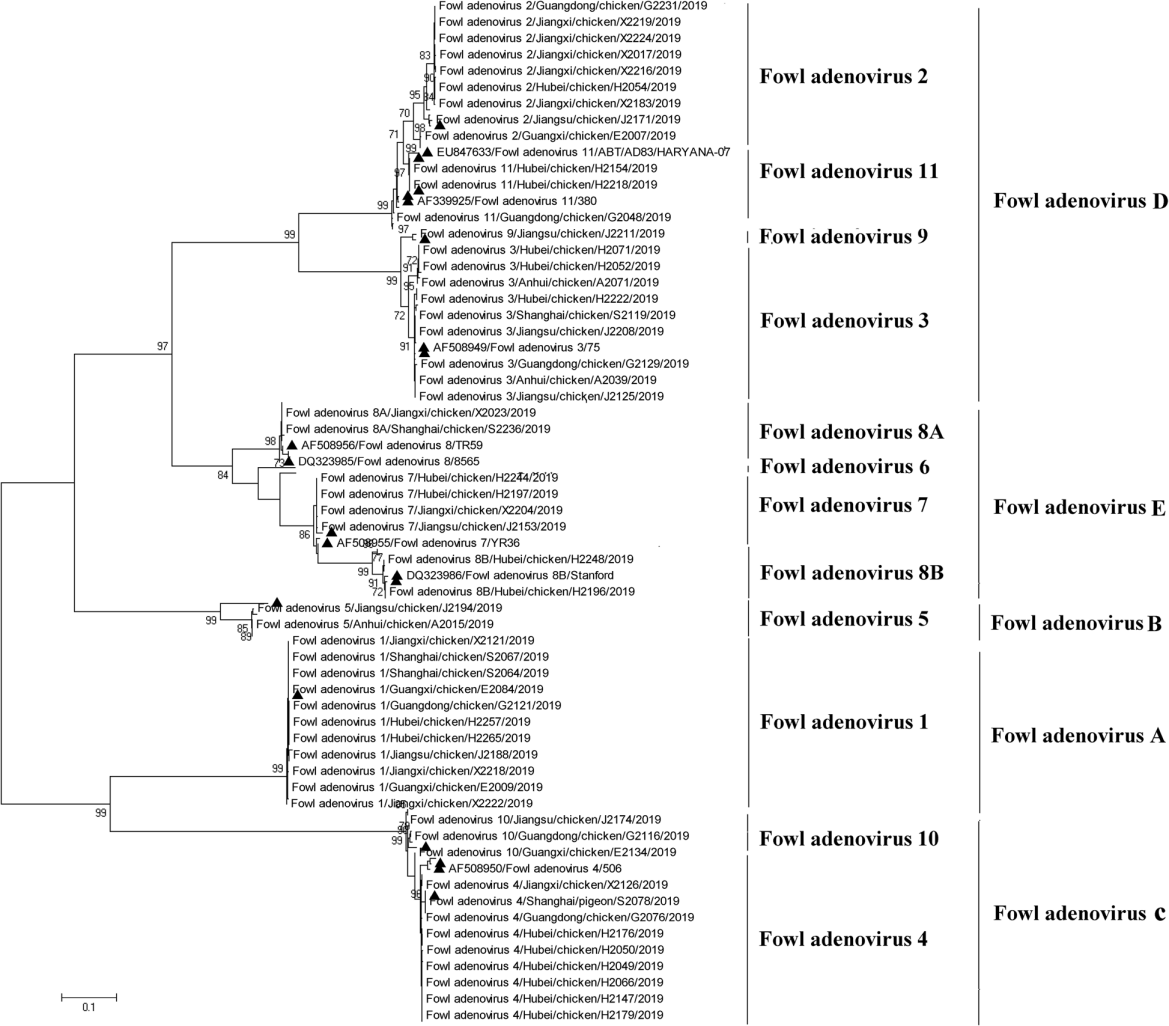
Phylogenetic analysis of FAdVs detected in this study and reference strains. Phylogenetic relationships were calculated using the model with the Maximum likelihood (ML). Gaps were handled by pairwise deletion and bootstrap values were calculated from 1, 000 replicates. FAdV strains in this study were marked with “▲”

The results of the phylogenetic analysis of FAdVs detected in this study and reference strains are shown in Fig. 2. In the analysis, the FAdV strains clustered into five major groups. Cluster 1 is corresponding to *Fowl aviadenovirus D* (*FAdV-D*), including serotypes 2, 3, and 11 FAdVs. Cluster 2 is corresponding to *Fowl aviadenovirus E* (*FAdV-E*), including serotypes 6, 7, 8A and 8B FAdVs. Cluster 3 is corresponding to *Fowl aviadenovirus B* (*FAdV-B*), including serotype 5 FAdVs. Cluster 4 is corresponding to *Fowl aviadenovirus A* (*FAdV-A*), including serotype 1 FAdV. Cluster 5 is corresponding to *Fowl aviadenovirus C* (*FAdV-C*), including serotypes 4 and 10 FAdVs. The phylogenetic analysis result in this study was consistent with the previous studies [4, 14, 16].

## Discussion

FAdVs are commonly present in fowl farms worldwide [17]. As reported, more and more FAdVs have been isolated from dead or sick animals in recent years [2, 6, 18–20]. FAdV infections are associated with a range of avian infectious diseases, such as IBH and HHS.

In China, since 2015, sporadic outbreaks of HHS occurred with suddenly high mortality rates in layers in most areas in China [7]. It was previously reported that the FAdV infection was caused by a variety of different FAdV species in China, at least two or three species of FAdVs (*species C, D or E*) were detected [7, 11]. In our survey, the present epidemiology surveillance showed more abundant diversity and wider distribution than the previous reported studies in China [7, 12, 14], the surveillance showed that all the five species (*species A, B, C, D and E*) FAdVs were detected in the FAdV survey in 7 provinces (Jiangxi, Shanghai, Guangxi, Jiangsu, Guangdong, Anhui, Hebei) of China. Significantly, all the detected samples in this survey were collected from apparently healthy birds, no diseased or dead fowls were sampled. This is a comprehensive survey from apparently healthy fowls. It has great significance for the prevention and control of circulation of fowl adenovirus in China.

Among all the circulating FAdV species, FAdV-D showed the highest percentage of 38.55% (including serotypes 2, 3, 9, and 11) in this study, which is different with the previous study [4, 11]. In this study, FAdV-D strains mainly circulating in Southern provinces of China, which including Anhui, Guangdong, Guangxi, Hubei, Jiangsu, Jiangxi, and Shanghai. Meanwhile, the prevalence rate of FAdV-A, FAdV-C and FAdV-E is maintained between 18.07% and 21.69% in southern China. Although the prevalent rate of species FAdV-A, FAdV-C and FAdV-E was lower than the dominant species FAdV-D, the strict biosecurity measures may be necessary to the prevention and control of FAdVs.

In the previous study, FAdV-1 [1], FAdV-2 [15], FAdV-4 [11], FAdV-7 [4], FAdV-8a [11], FAdV-8b [11], FAdV-10 [15], and FAdV-11 [14] have been reported in China. In this study, 166 samples (166/1920, 8.65%) were detected FAdV positive in the 1920 clinical samples, and all the FAdVs were detected from LBMs, the results suggest that biosafety measures should be strengthened in the LBMs. Totally all the FAdV serotypes of 1, 2, 3, 4, 5, 6, 7, 8A, 8B, 9, 10 and 11 were detected. Slightly different from previous reports [4, 11], the most prevalent serotype in this study was serotype 1 (30/166, 18.07%), not serotype 4 [11], FAdV-11 [14] or FAdV-8a [11], FAdV-8b [11]. Of course, serotypes 2, 3 and 4 remained at a relatively high prevalence rate of 15.66%, 16.27% and 16.87%, respectively. This difference may be due to the fact that FADVs are screened from apparently healthy birds, and the published articles are from diseased birds. The prevalence rate of other serotypes of FAdVs was below 7% in China in this study. In this study, the circulating serotypes showed abundant diversity and distributed in 8 provinces in China, this increased the difficulty for the prevention and control of the FAdVs.

The results showed that all the FAdV-D strains and the most prevalent serotype 1 strains were detected from chickens in LPMs, no positive samples were detected from slaughterhouse or large-scale farms. This indicated that chicken may be an important risk, which indicated that chickens in the LPMs of southern China may play an important role in the transmission and circulation of FAdVs. According to the relevant reports around the world, duck adenovirus have been reported since 2014 [21–26], and infection of FAdV-4 in geese and pigeon adenovirus 1 have been reported in the previous study [27–29]. Meanwhile, FAdVs were also detected from ducks, geese and pigeons in this study. The role of waterfowl and birds (e.g. pigeon) in the spread of FAdVs should be paid more attention in the further study. All the cloacal/throat double swabs in this study were collected from clinical healthy birds according to a random sampling method. The results showed that 8.65% collected samples were positive in the FAdV detection. As reported, it is believed that the mechanism of FAdV infection is very complex in chicken flocks [7]. The pathogenicity, such as FAdV-D and FAdV-E, was not evaluated and, thus, further investigation are warranted.

## Conclusion

Taken together, in the FAdV epidemiological survey, 8.65% of the clinical samples from apparently healthy birds were positive in the fowl adenovirus PCR detection. Totally all the 12 serotypes fowl adenoviruses were detected in a variety of fowl species, which provided abundant resources for the research of fowl adenoviruses in China. The present study provides new information about the epidemiology and characteristics of fowl adenoviruses associated with chickens, ducks and pigeons in China, which will provide a basis for further understanding of the disease, and would aid in the prevention and control of FAdVs. Surveillance for fowl adenovirus must be continued worldwide.

## Materials and methods

### Sample collection and Nucleic acid extraction

In 2019, totally 25 sites of large-scale farms, slaughterhouse and LBMs of the 8 provinces (Hubei, Jiangxi, Shanghai, Guangxi, Jiangsu, Guangdong, Anhui, Hebei) in China were selected at random. Cloacal/throat double swabs were collected from more than 60 clinical healthy birds at each farm according to a random sampling method. Totally, 1920 swab samples were collected, the sample details were listed in Table 4. The samples were stored at 4 °C by a preservation buffer of 1.2 mL phosphate buffered saline (PBS, pH 7.2) containing 10% glycerol. Based on the QIAxtractor platform, viral DNA was extracted using the cador Pathogen 96 QIAcube HT kit (Qiagen, Hilden, Germany) according to the manufacturer’s instructions, and stored at − 80 °C.

**Table 4:** The details of 1920 samples collected in this study

Fowl	LBM	Slaughterhouse	Poultry flock	Total
goose	40	0	0	40
pigeon	235	0	0	235
chicken	831	240	150	1221
duck	419	0	0	419
partridge	5	0	0	5
**total**	**1530**	**240**	**150**	**1920**

### Screening of FAdV in clinical samples

In order to screen and analyze FAdV, conventional PCR using degenerate primers specific for the L1 loop of the FAdV hexon genes was applied according to the published report [30]. The primers were designed by alignment comparison of the conserved sequences of FAdV1, FAdV8 and FAdV9, and were able to amplify the 12 serotypes of FAdVs [30]. A 897-base fragment of hexon gene was amplified using primers: hexon A (forward, in the position 144–161 of FAdV1 with the GenBank accession number of U46933) 5′- CAARTTCAGRCAGACGGT-3′ and hexon B (reverse, in the position 1229–1211 of FAdV1 with the GenBank accession number of U46933) 5′- TAGTGATGMCGSGACATCAT -3′. The DNA was tested by PCR with the Premix Taq™ (Ex Taq™ Version 2.0) (Takara, Dalian, China; RR003Q), and the reaction system included 12.5 μL of Premix Taq, 1 μL each of the forward and reverse primers (10 pM), 3 μL of DNA, and 7.5 μL of RNase-free H_2_O. The thermocycling conditions were as follows: 95 °C for 5 min, followed by 40 cycles of 98 °C for 30 s, 60 °C for 30 s and 72 °C for 60 s, followed by a final extension for 10 min at 72 °C. The gene fragment PCR results were analyzed using a QIAxcel DNA Screening Kit (2400) (Qiagen) using the QIAxcel Advanced System.

As reported in the previous studies [4, 14, 16], it is shown that the amplification of the Hexon gene by molecular biology method can also be used to identify the serotype of FAdVs. We identified the serotype of FAdVs by using molecular biology methods in this study.

### Sequencing

PCR products were purified using a QIAquick PCR purification kit (Qiagen, Hilden, Germany), cloned into the pMD18-T vector (Takara, Dalian, China), and then sequenced using synthetic oligonucleotides (Sangon Biotech, Shanghai, China). The sequences were submitted to the GenBank database, National Center for Biotechnology Information.

### Phylogenetic analysis

All the hexon sequences derived from the detected FAdV were aligned with 22 selected hexon genes of the 12 serotypes FAdV reference strains in the ICTV classification system and some other selected field strains, following the published reports [30]. Hexon gene sequences of the representative strains were downloaded from NCBI, GenBank accession numbers of these sequences were listed in Table 5. The phylogenetic tree based upon the results of multiple sequence alignment was constructed using the Molecular Evolutionary Genetics Analysis (MEGA) software version 7.0 [31, 32] applying the model with the maximum likelihood (ML) method, The robustness of the phylogenetic constructions was evaluated by bootstrapping with 1,000 replicates [31]. Serotypes were identified based on the phylogenetic analysis and pairwise identities as described in previous studies [30].

**Table 5:** FAdV reference strains and field strains corresponding to the 12 serotypes in the International Committee on Taxonomy of Viruses classification system

Strain	Serotype	Accession number	Reference publication
Phelps(ATCCVR-432)	FAdV-1	NC001720	[33]
685	FAdV-2	AF508947	[34]
SR49	FAdV-3	AF508948	[34]
75	FAdV-3	AF508949	[34]
506	FAdV-4	AF508950	[34]
SDSX	FAdV-4	KT899325	[35]
KR5	FAdV-4	AF508951	[34]
340	FAdV-5	KC493646	[36]
CR119	FAdV-6	KT862808	[37]
YR36	FAdV-7	AF508955	[34]
B-3A(ATCCCVR-832)	FAdV-7	AF339922	[34]
TR59	FAdV-8a	AF508956	[34]
8565	FAdV-8a	DQ323985	[38]
764	FAdV-8b	AF508958	[37]
Stanford	FAdV-8b	DQ323986	[38]
A-2A	FAdV-9	AC_000013	[5]
C-2B	FAdV-10	KT717889	[37]
380	FAdV-11	AF339925.1	[34]
1047	FAdV-11	DQ323984	[38]
ABT/AD83/HARYANA-07	FAdV-11	EU847633	[24]
C2B	FAdV-11	AF508959.2	[34, 35]
ON P2	FAdV-11	KU310942	[39, 40]

## Data Availability

The datasets generated and/or analyzed during the current study are available in the the GenBank database of National Center for Biotechnology Information, persistent web link is https://www.ncbi.nlm.nih.gov/, the GenBank accession numbers of FAdVs are ON502449-ON502604 and ON462358-ON462367.

## Abbreviations

FAdV: Fowl adenovirus
LBMs: Living bird markets
